# Supplementary material for: cloneXplorer: A high-throughput clone discovery platform based on conical microwell arrays
Source: bioRxiv. 2026 Jan 20:2026.01.16.699323. Preprint. [Version 1] doi: 10.64898/2026.01.16.699323 (PMC12871585; doi:10.64898/2026.01.16.699323)
Supplement: 1 [file NIHPP2026.01.16.699323V1-supplement-1.pdf]

bioRxiv preprint doi: https://doi.org/10.64898/2026.01.16.699323; this version posted January 20, 2026. The copyright holder has placed this preprint (which was not certified by peer review) in the Public Domain. It is no longer restricted by copyright. Anyone can legally share, reuse, remix, or adapt this material for any purpose without crediting the original authors.

Supplementary Analysis of Co-Culture Interaction Combinations in Microwells

We aim to estimate the maximum number of TCR/epitope combinations that can be screened in one experiment. Let **N** be the number of microwells filled with an average occupancy, **Λ**, cells per microwell, such that $\mathbf{M = N\,\Lambda}$ is the total number of cells detected in the observed microwells. The total cell input consists of two populations: $\mathbf{N\,\Lambda = N\,(\Lambda_1 + \Lambda_2)}$, where $\mathbf{\Lambda_1}$ is the average occupancy of the TCR-expressing Jurkat cells and $\mathbf{\Lambda_2}$ is the average occupancy of the antigen-presenting K562 cells (APCs). Assuming each individual **J** species in the TCR-expressing population is equally represented as $\mathbf{\lambda_j = \Lambda_1 / J}$ for $j \in \{1 .. J\}$ and similarly for the **K** species of APCs with probabilities as $\mathbf{\lambda_k = \Lambda_2 / K}$ for $k \in \{1 .. K\}$, then the joint probability of finding a j/k pair in any given microwell is $\mathbf{\lambda_{jk} = (\Lambda_1 / J)\,(\Lambda_2 / K)}$. The ability to observe each j/k pair requires enough microwells to satisfy: $\mathbf{\alpha\,N\,\lambda_{jk} >> 1}$, where $\alpha \in [0,1]$ is the probability that a positive signal is observed in a microwell that contains a matching TCR/antigen pair and is ideally close to unity. In this work, we found that $\alpha \sim 0.7$, meaning that GFP+ microwells are observed in 70% of instances where there is a matching TCR/antigen pair. With substitution, we can estimate the upper limit of the assay as: $\mathbf{\alpha\,N\,(\Lambda_1 / J)(\Lambda_2 / K) >> 1}$.

To evaluate scaling trends, assume $\mathbf{J \sim K}$ and $\mathbf{\Lambda_1 \sim \Lambda_2}$, implying an equal number of Jurkat and K562 cells are loaded into the microwells, and their libraries have equal plexity. This yields $\mathbf{P < \Lambda^2\,(\alpha\,N / 4)}$, where **P** is the maximum plexity for given values of **Λ** and **N**. This relationship shows that the optimal plexity increases linearly with the number of cells loaded, which makes sense because the number of interactions occurring in microwells scales quadratically with the number of cells inside. The plexity depends linearly on the number of microwells. For example, if there are **N = 270,000** pickable microwells and with $\mathbf{\Lambda \sim 2}$ and $\mathbf{\alpha \sim 1}$, a library of size ~165x165 will yield ~10 co-culture pairs for each J/K combination. Since the recovered sequences are derived from small cell populations of ~ 2 – 5 cells, the correctly matched j/k pairs must occur at sufficient frequency to offset counts from bystander sequences recovered from those microwells. To validate the interaction, the j/k pairs must be observed in the sequences recovered from at least 3 independent microwells.

This analysis suggests the possibility of screening millions of TCR/antigen pairs simultaneously by loading the microwells with dozens of cells per well. However, this throughput can only be achieved by reducing background noise caused by randomly activated T cells, which pollute the deconvolution pipeline. Background noise can be reduced by pre-sorting the Jurkat population with a GFP-negative gate, or better yet, by identifying the pre-activated T cells during live cell imaging. The ability to image microwells at multiple time points allows false positives to be eliminated, which is an advantage over systems that rely on single time point imaging or endpoint genomic assays. If background can be reduced below 0.001% using a gating scheme based on multiple timepoints and functional parameters, then it should be possible to screen 100,000 TCR/epitope interactions in one plate. This platform can handle both single library screens, such as one TCR evaluated against a pool of 100,000s of antigens, as well as library-on-library screens, such as the validation of a short list of 100 TCR candidates against a smaller library of 1,000 antigens.

bioRxiv preprint doi: https://doi.org/10.64898/2026.01.16.699323; this version posted January 20, 2026. The copyright holder has placed this preprint (which was not certified by peer review) in the Public Domain. It is no longer restricted by copyright. Anyone can legally share, reuse, remix, or adapt this material for any purpose without crediting the original authors.

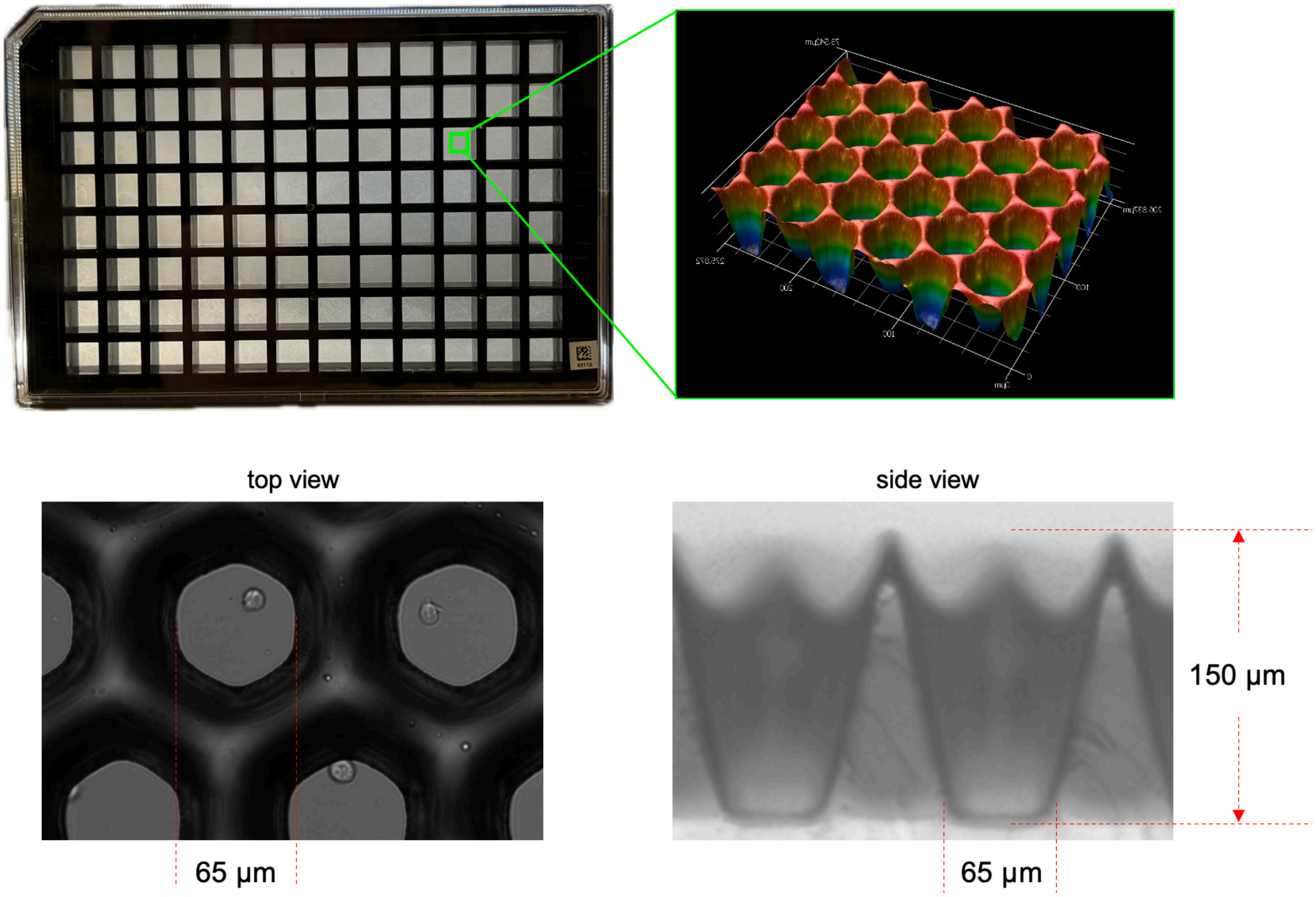

Supplementary Fig. 1. (Top Left) A photograph of the microwell plate. (Top Right) A magnified view of the microwells measured by optical profilometry. (Bottom Left) A top view of microwells loaded with cells. (Bottom Right) A side view of the microwells.

bioRxiv preprint doi: https://doi.org/10.64898/2026.01.16.699323; this version posted January 20, 2026. The copyright holder has placed this preprint (which was not certified by peer review) in the Public Domain. It is no longer restricted by copyright. Anyone can legally share, reuse, remix, or adapt this material for any purpose without crediting the original authors.

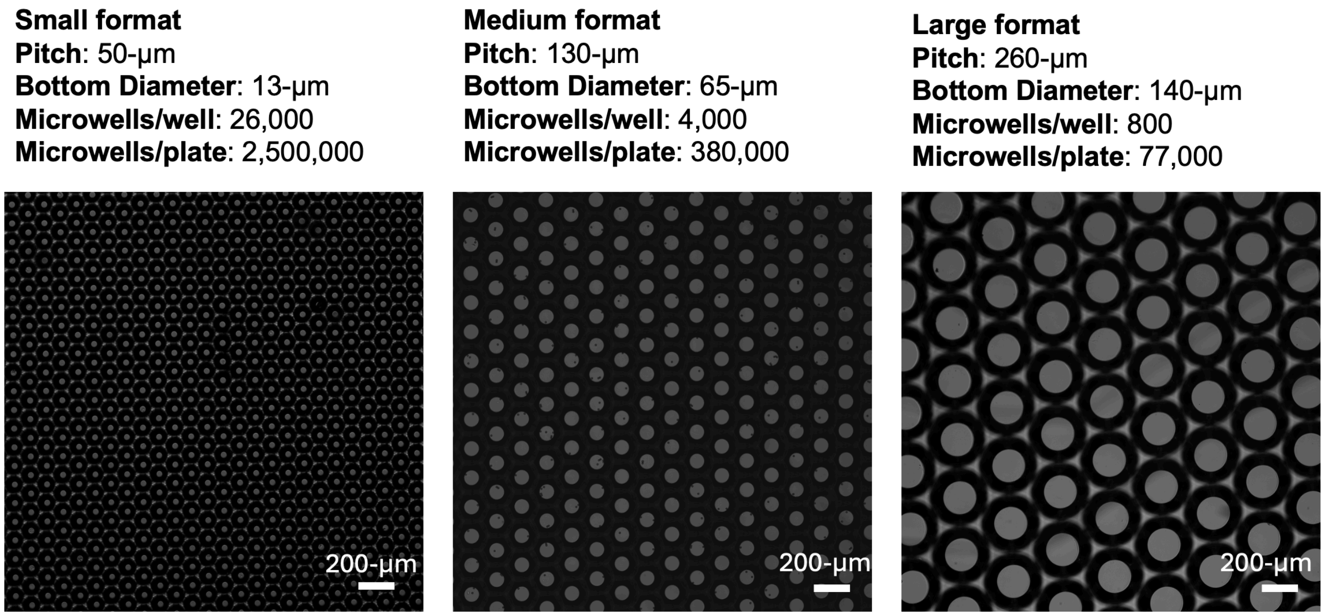

Supplementary Fig. 2. Comparison of microwells with different sizes that Celldom manufactures for different applications. The medium format is used in this work.

bioRxiv preprint doi: https://doi.org/10.64898/2026.01.16.699323; this version posted January 20, 2026. The copyright holder has placed this preprint (which was not certified by peer review) in the Public Domain. It is no longer restricted by copyright. Anyone can legally share, reuse, remix, or adapt this material for any purpose without crediting the original authors.

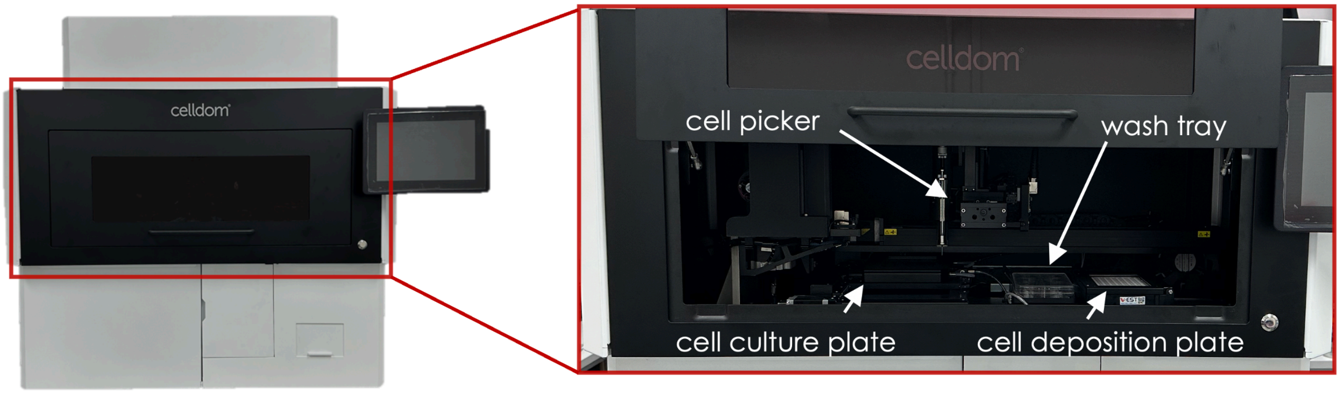

Supplementary Fig. 3. cloneXplorer instrument with closed door (left), and open door (right).

bioRxiv preprint doi: https://doi.org/10.64898/2026.01.16.699323; this version posted January 20, 2026. The copyright holder has placed this preprint (which was not certified by peer review) in the Public Domain. It is no longer restricted by copyright. Anyone can legally share, reuse, remix, or adapt this material for any purpose without crediting the original authors.

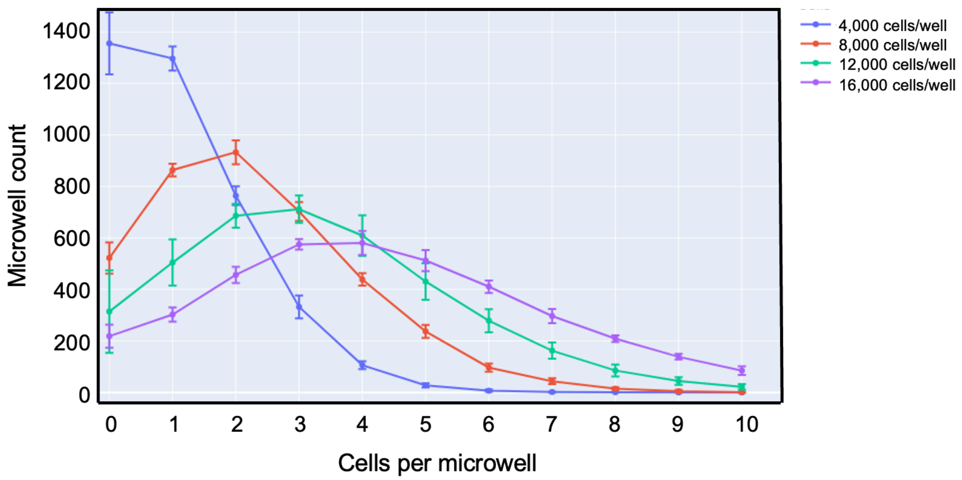

Supplementary Fig. 4. Cell loading distribution for 4,000, 8,000, 12,000, and 16,000 cells seeded per well.

bioRxiv preprint doi: https://doi.org/10.64898/2026.01.16.699323; this version posted January 20, 2026. The copyright holder has placed this preprint (which was not certified by peer review) in the Public Domain. It is no longer restricted by copyright. Anyone can legally share, reuse, remix, or adapt this material for any purpose without crediting the original authors.

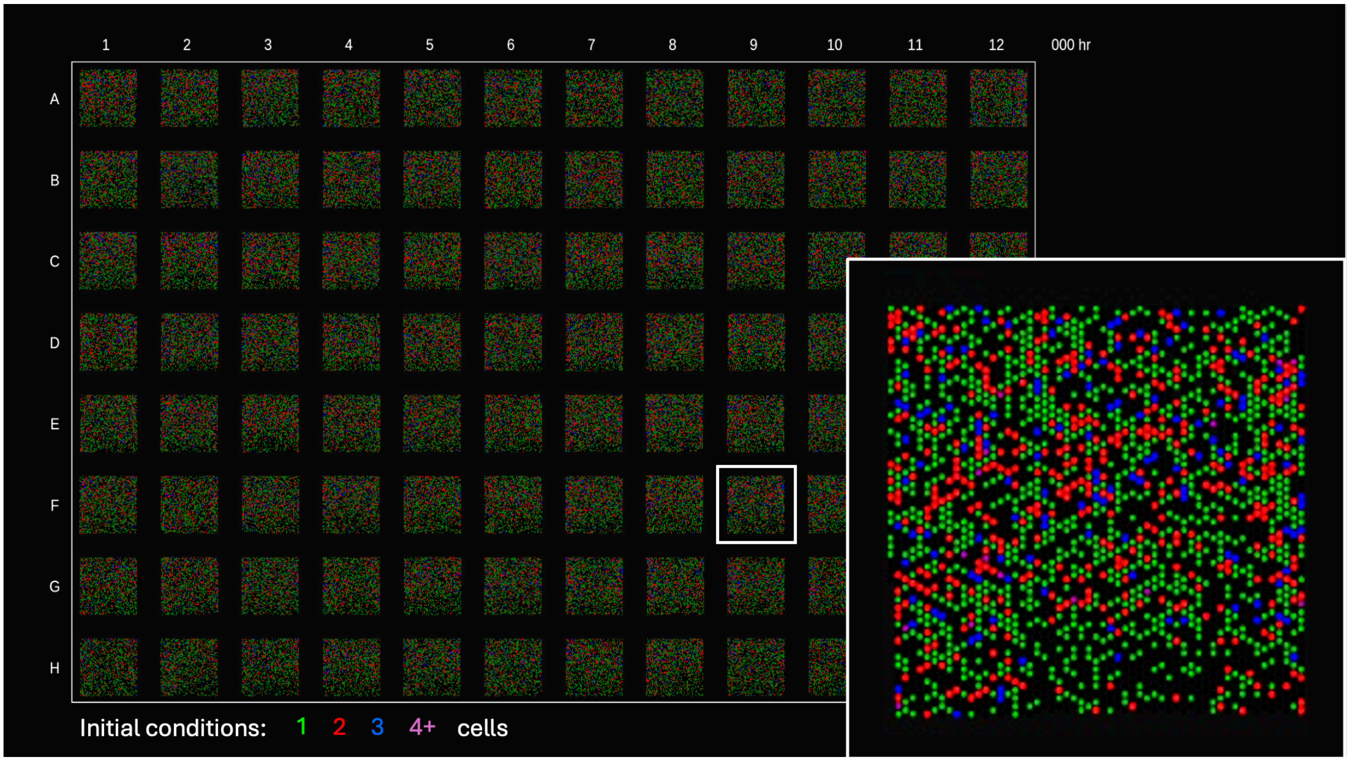

Supplementary Fig. 5. Cell loading distribution for 3,300 cells per well. The black, green, red, blue, and purple colors indicate microwells that contain 0, 1, 2, 3, or 4+ cells.

bioRxiv preprint doi: https://doi.org/10.64898/2026.01.16.699323; this version posted January 20, 2026. The copyright holder has placed this preprint (which was not certified by peer review) in the Public Domain. It is no longer restricted by copyright. Anyone can legally share, reuse, remix, or adapt this material for any purpose without crediting the original authors.

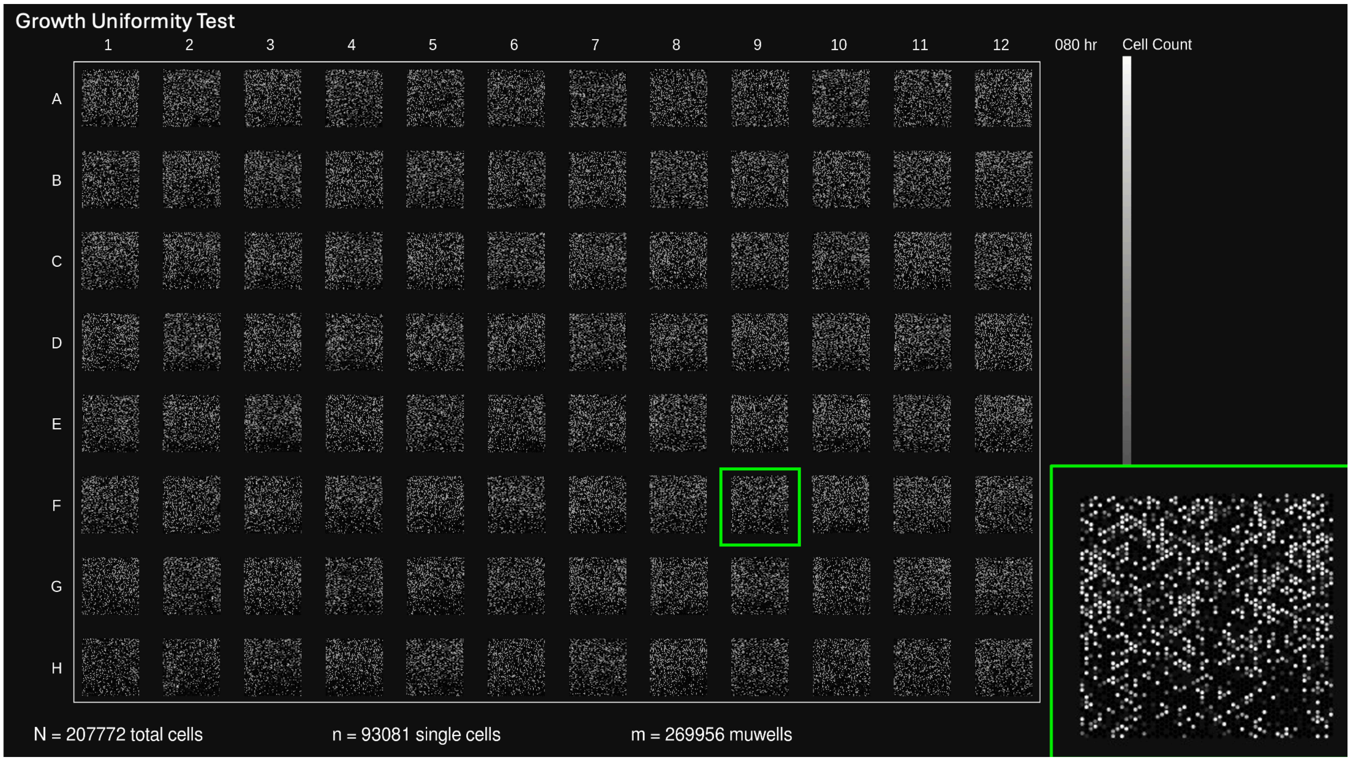

Supplementary Fig. 6. In the microwells that initially had a single cell, the cell count map is shown at 80 hours with grayness level proportional to the number of cells. The whitest color is 10 cells.

bioRxiv preprint doi: https://doi.org/10.64898/2026.01.16.699323; this version posted January 20, 2026. The copyright holder has placed this preprint (which was not certified by peer review) in the Public Domain. It is no longer restricted by copyright. Anyone can legally share, reuse, remix, or adapt this material for any purpose without crediting the original authors.

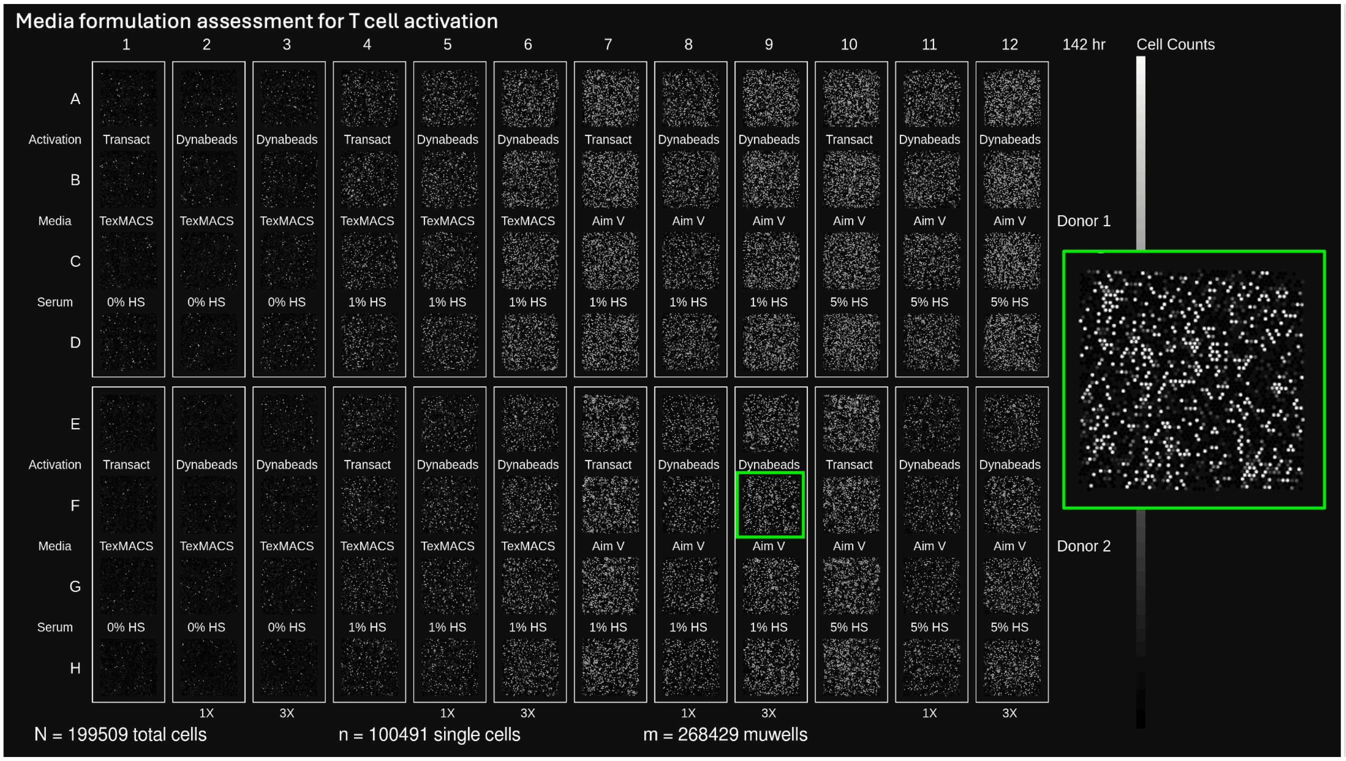

Supplementary Fig. 7. Cell growth distribution for freshly thawed T cells exposed to different levels of human serum (0%, 1%, 5%), different methods of CD3/CD28 stimulation (Dynabeads vs Transact), and different media conditions (TexMACS vs AIM V). The gray level indicates the cell count at 142 hr on a scale of 0 (black) to 100 (white).

bioRxiv preprint doi: https://doi.org/10.64898/2026.01.16.699323; this version posted January 20, 2026. The copyright holder has placed this preprint (which was not certified by peer review) in the Public Domain. It is no longer restricted by copyright. Anyone can legally share, reuse, remix, or adapt this material for any purpose without crediting the original authors.

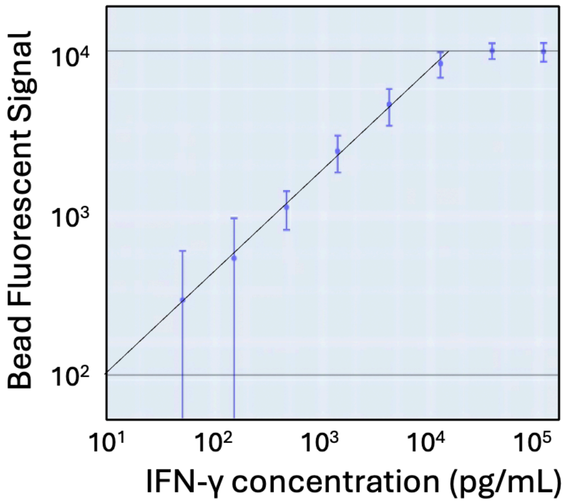

Supplementary Fig. 8. Fluorescent signal from IFN-γ capture beads in microwells exposed to different concentrations of soluble cytokine IFN-γ.

bioRxiv preprint doi: https://doi.org/10.64898/2026.01.16.699323; this version posted January 20, 2026. The copyright holder has placed this preprint (which was not certified by peer review) in the Public Domain. It is no longer restricted by copyright. Anyone can legally share, reuse, remix, or adapt this material for any purpose without crediting the original authors.

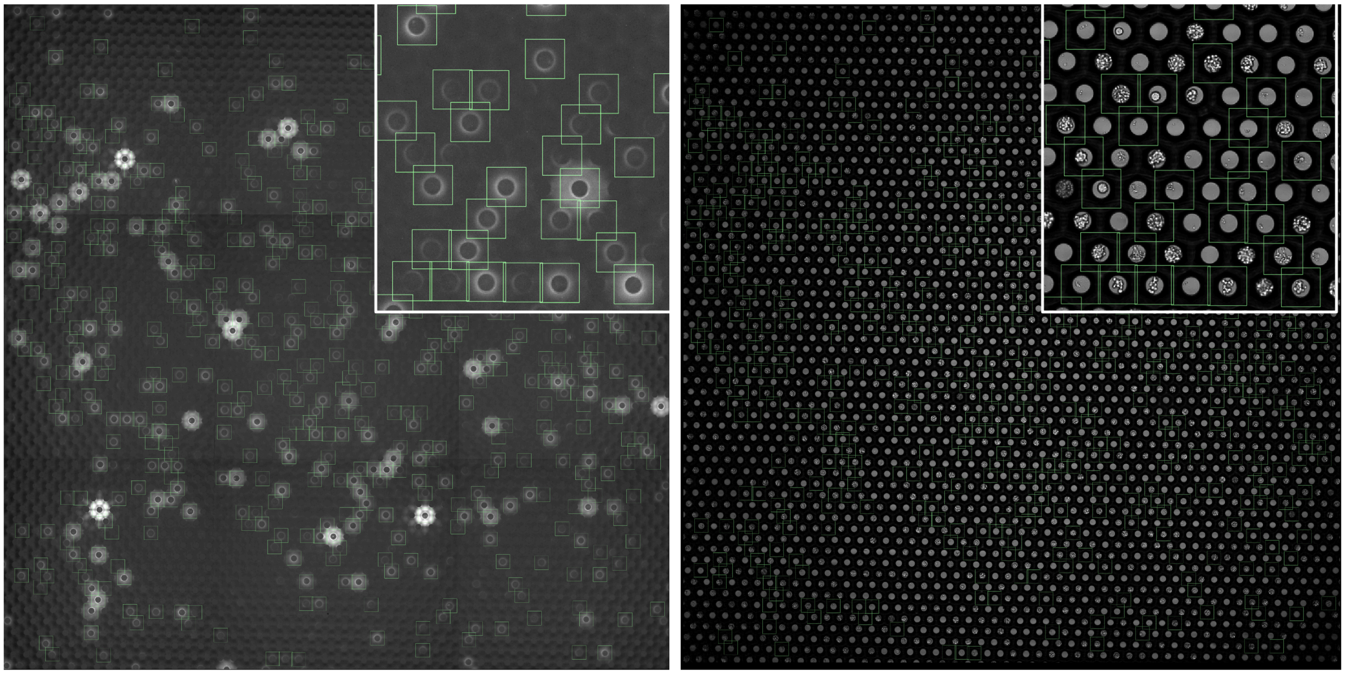

Supplementary Fig. 9. Whole well stitch of IFN-γ secretion pattern (left) and the corresponding brightfield images (right) in a coculture of mouse OT-1 cells and MC-38 cells presenting SIINFEKL peptide.

bioRxiv preprint doi: https://doi.org/10.64898/2026.01.16.699323; this version posted January 20, 2026. The copyright holder has placed this preprint (which was not certified by peer review) in the Public Domain. It is no longer restricted by copyright. Anyone can legally share, reuse, remix, or adapt this material for any purpose without crediting the original authors.

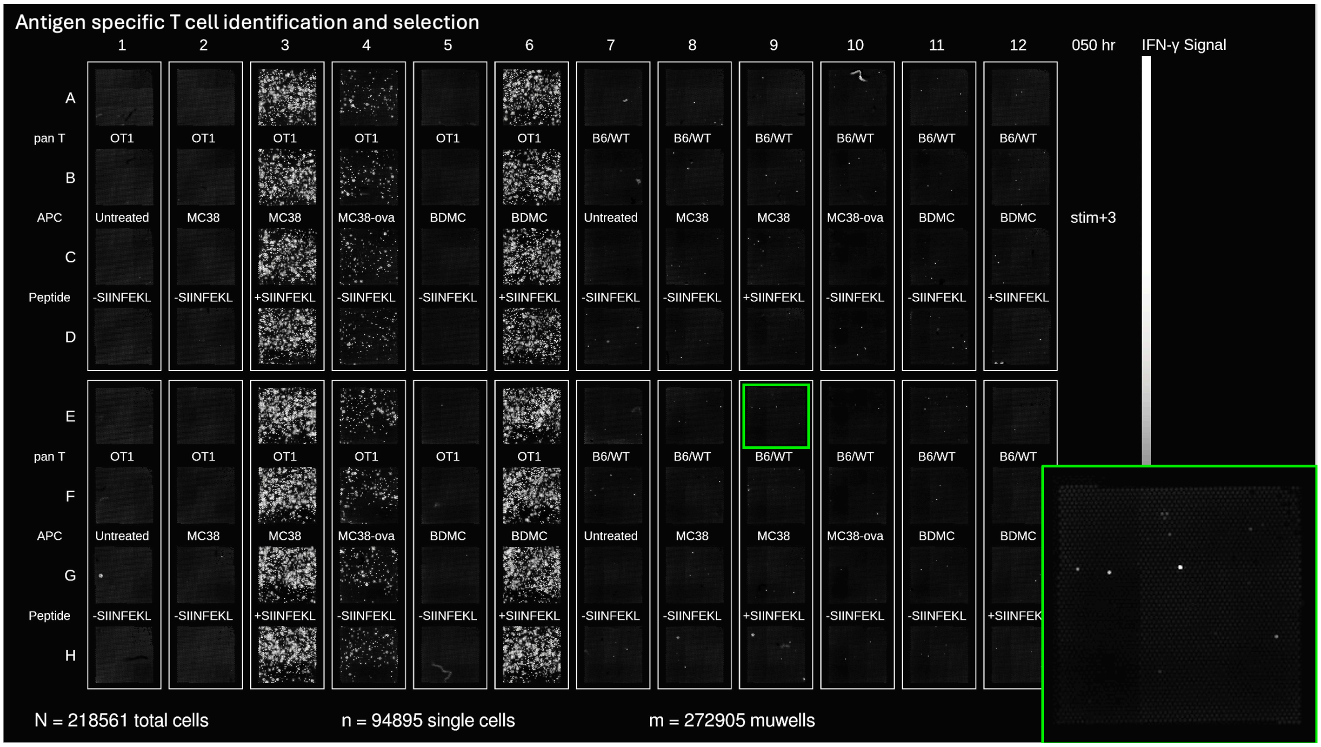

Supplementary Fig. 10. A microwell map of IFN-γ secretion at 50 hours is shown for co-cultures of splenocyte derived mouse T cells (OT-1 vs B6/WT) in co-culture with different antigen presenting cells (MC38, MC38-ova, BMDC) in the presence or absence of SIINFEKL peptide.

bioRxiv preprint doi: https://doi.org/10.64898/2026.01.16.699323; this version posted January 20, 2026. The copyright holder has placed this preprint (which was not certified by peer review) in the Public Domain. It is no longer restricted by copyright. Anyone can legally share, reuse, remix, or adapt this material for any purpose without crediting the original authors.

	**Bruker**	**Cell Microsystems**	**Sartorius**	**Celldom**
Instrument	Beacon	CellRaft Air	CellCelector	cloneXplorer
Consumable	OptoSelect 3500	CellRaft	Flex S200	cloneXpress
Number of samples	4	24	6 or 24	96
Partitions per experiment	14,000	154,000	100,000	380,000
Single cell occupancy	> 80%	< 40%	< 40%	< 40%
Partition aspect ratio (H/W)	> 10	0.5	0.5	> 2
Loading Time	1 hr	10 min	10 min	10 min
Environment control	Yes	No	No	Yes
Media exchange	Yes	No	No	Yes
Small molecule drug screens	Yes	No	Yes	Yes
Reference	Ref. 19	Ref. 12	Ref. 13	**This work**

Table 1. Comparison of commercial systems capable of analyzing cell function at multiple time points with ability to retrieve clones of interest for applications in cell line development and immune discovery.

bioRxiv preprint doi: https://doi.org/10.64898/2026.01.16.699323; this version posted January 20, 2026. The copyright holder has placed this preprint (which was not certified by peer review) in the Public Domain. It is no longer restricted by copyright. Anyone can legally share, reuse, remix, or adapt this material for any purpose without crediting the original authors.

	**Specifications**
Consumable	96 well plate (SLAS)
Imaging Fields (9 per well)	864
Image pixel size	0.5 µm
Full plate scan BF only	15 min
Full plate scan BF + 4-FL channels	30 min
Autofocusing	every imaging field
Temperature Control	+/-0.2ºC
Pickable microwells per well	2,800
Total microwells per well	4,000
Humidity Control	>90% R.H.
CO_2 control	0 – 10%
O_2 control	0 – 20%
Full plate image analysis	60 min
Picking time without rinsing	30 seconds
Picking time with rinsing	2 - 5 minutes
Picking success rate	>90%

Table 2. Datasheet for the cloneXplorer

bioRxiv preprint doi: https://doi.org/10.64898/2026.01.16.699323; this version posted January 20, 2026. The copyright holder has placed this preprint (which was not certified by peer review) in the Public Domain. It is no longer restricted by copyright. Anyone can legally share, reuse, remix, or adapt this material for any purpose without crediting the original authors.

SUPPLEMENTARY MOVIE LEGENDS

MOVIE S1. 7 μm fluorescent beads are retrieved from microwells in 250 successive picks.

MOVIE S2. NALM6-GFP cells are retrieved from microwells in 35 successive picks.

MOVIE S3. A movie of a single K562 cell imaged at 4 hour intervals for 56 hours is shown with and without the segmentation masks.

MOVIE S4. To assess growth rate uniformity across a 96-well plate, the NALM6 cell count is shown at 8 hour intervals for 80 hours. Out of the 207,772 cells identified at the initial timepoint, 93,081 were single cells.

MOVIE S5. To assess uniformity in the proliferation rates of primary T cells, freshly thawed T cells were exposed to Miltenyi Transact in AIM V media supplemented with 5% human serum and imaged at 3 hour intervals for 130 hours.

MOVIE S6. To assess uniformity in the proliferation rates of primary T cells, freshly thawed T cells were exposed to ThermoFisher Dynabeads in AIM V media supplemented with 5% human serum and imaged at 3 hour intervals for 130 hours.

MOVIE S7. The activation dynamics of freshly thawed human T cells exposed to different CD3/CD28 activation matrices (Miltenyi Transact vs ThermoFisher Dynabeads) in different media (AIM V vs TexMACS) and supplemented with different levels of human serum (0%, 1%, 5%) is shown as a map of cell counts imaged at 3 hour intervals for 130 hours. Out of the 199,509 cells identified at the initial timepoint, 100,491 were single cells.

MOVIE S8. The life of a single cell from the day of seeding through 3 days of expansion is shown, followed by documentation of the cell picking process, including images taken before and after cell isolation in brightfield and fluorescent channels.

MOVIE S9. Time lapse brightfield images are shown for co-cultures of mouse T cells (OT-1) and MC-38 ova cells.

MOVIE S10. The characteristic IFN-γ secretion patterns are shown for co-cultures of mouse T cells (OT-1) and MC-38 ova cells at 4-hour intervals for 50 hours.

MOVIE S11. A secretion map of mouse T cells (OT1 vs B6/WT) in co-culture with antigen presenting cells (MC38, MC38-ova, BMDC) and exposed to SIINFEKL peptide is shown at 4 hour intervals for 50 hours. Out of the 218,561 cells identified at the initial timepoint, 94,895 were single cells.
